# Compulsory Subscriptions

**Published:** 1910-03

**Authors:** 


### Compulsory Subscriptions.

Some of our subsidized contemporaries, who are striving to pay their paper and printer's bills by slipping under the canvas of a State or National society, have been puzzling their heads as to how to comply with the postal laws. The Postoffice Department has ruled that there must be an individual subscription for each subscriber who receives a publication that enjoys second-class mail privileges. But the law is not complied with by a person paying two dollars a year to the State society, and then by some hocus pocus receive a periodical publication for nothing. The same with the national organ of the A. M. A. It would seem that if the subscription to the State society is one dollar and the subscription to a journal is one dollar, making a total of two dollars, the member should be allowed, if he desires it, to pay for the membership alone, and not take the journal, if he feels that he does not need it. So with the national journal. Everyone admits that it is a good four dollars' worth, but the management publishes the notice that "the annual assessment for membership shall be one dollar; and the price of subscription to the *Journal* shall be four dollars for members." This being the case, one fails to see why, if one desires to be a member of the association alone, he should not be allowed to pay his dollar. Then he can do as he pleases about the *Journal.* Perhaps he may wish to commit the unpardonable sin of reading the journal that comes to his partner. If so, that should be no one's business but that of himself and his partner.

The entire question is—shall an intelligent body of men be dragooned, by a subterfuge, into subscribing in a roundabout way, for a journal that for some good reason they do not care to pay for? We may try to get a youthful patient to take a pill by sugar coating it, or by putting it in a spoonful of jam. But that is no reason why the members of the medical profession should have their societies used as a compulsory canvassing machine, requiring the member, willy nilly, to pay for a journal.—*Medical Sentinel.*

